# Atrial cardiopathy and cognitive impairment

**DOI:** 10.3389/fnagi.2022.914360

**Published:** 2022-07-22

**Authors:** Sarah J. Myers, Amado Jiménez-Ruiz, Luciano A. Sposato, Shawn N. Whitehead

**Affiliations:** ^1^Department of Anatomy and Cell Biology, Schulich School of Medicine and Dentistry, Western University, London, ON, Canada; ^2^Department of Clinical Neurological Sciences, University Hospital, Western University, London, ON, Canada

**Keywords:** heart failure, cognitive impairment, atrial cardiopathy, dementia, atrial fibrillation

## Abstract

Cognitive impairment involves complex interactions between multiple pathways and mechanisms, one of which being cardiac disorders. Atrial cardiopathy (AC) is a structural and functional disorder of the left atrium that may be a substrate for other cardiac disorders such as atrial fibrillation (AF) and heart failure (HF). The association between AF and HF and cognitive decline is clear; however, the relationship between AC and cognition requires further investigation. Studies have shown that several markers of AC, such as increased brain natriuretic peptide and left atrial enlargement, are associated with an increased risk for cognitive impairment. The pathophysiology of cognitive decline in patients with AC is not yet well understood. Advancing our understanding of the relationship between AC and cognition may point to important treatable targets and inform future therapeutic advancements. This review presents our current understanding of the diagnosis of AC, as well as clinical characteristics and potential pathways involved in the association between AC and cognitive impairment.

## Introduction

**FIGURE 1:**
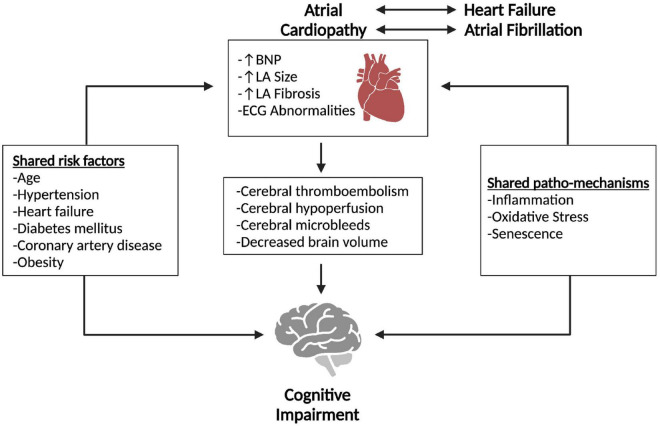
Overview of proposed relationship between atrial cardiopathy and cognitive impairment. Atrial cardiopathy, atrial fibrillation and heart failure all share multiple risk factors and patho-mechanisms with cognitive impairment. The effects of atrial cardiopathy potentially predispose individuals to cognitive impairment. Pathways that lead from atrial cardiopathy to cognitive impairment may include cerebral thromboembolism, hypoperfusion, microbleeds, and brain volume loss. Created with Biorender.com.

Atrial cardiopathy (AC) is a recently described structural and functional disorder of the left atrium (LA). The lack of a standardized definition for this condition reflects the heterogeneous nature in the spectrum of disorders affecting the LA, resulting in an increased risk of thromboembolic events. Potential biomarkers of AC include imaging, electrophysiological, and serum abnormalities. AC may also be a substrate for multiple primary cardiac disorders, including atrial fibrillation (AF) and heart failure (HF), and has been linked to increased risk for stroke and higher stroke mortality (Ahmad et al., 2020; Edwards et al., 2020). While the link between AF and HF and cognition is clear, data on AC and cognition are scarce (Aldrugh et al., 2017; Rosler and Schnabel, 2020). This review summarizes the relevant anatomical and functional pathways involved in AC and the epidemiology, diagnosis, clinical characteristics, prognosis, and potential treatments for AC and cognitive impairment. An overview of the proposed relationship between AC and cognitive impairment is shown (Figure 1).

## Diagnostic criteria

A range of diagnostic criteria for AC has been proposed, including the use of echocardiography, electrocardiography, cardiac magnetic resonance imaging (MRI), and blood serum markers (Yaghi et al., 2017). An overview of these diagnostic criteria is provided (Table 1).

**TABLE 1:** Proposed diagnostic criteria for AC.

Diagnostic method	Metrics	AC criteria
Echocardiography	LA volume or volume index; LA diameter or diameter index; LA area or area index	↑ LA size ↓ LA flow velocity
Electrocardiography	PR Interval P-wave terminal force in V1	↑ PR interval ↑ P-wave terminal force in V1
Cardiac MRI	Uptake of gadolinium	↑ LA fibrosis (decreased uptake of gadolinium)
Blood serum biomarkers	BNP Levels Cardiac troponin levels	↑ BNP ↑ Cardiac troponin

Echocardiography can be used to evaluate LA diameter or volume, with LA enlargement being considered a marker of AC (Goldberger et al., 2015). The most reliable measure is the LA volume index, although it is more rarely reported (Healey et al., 2019; Kamel et al., 2019). LA enlargement is also associated with a higher rate of cardiovascular events and cardiovascular death (Menichelli et al., 2020). Other echocardiography markers of AC include spontaneous echocardiographic contrast and reduced LA appendage flow velocity (Yaghi et al., 2017). Both of which are also associated with an increased risk of thrombus formation (Leung et al., 1994; Lee et al., 2014).

Multiple electrocardiogram measures may be used as markers of AC, such as PR interval and P-wave terminal force in V1 (Cheng et al., 2009; Yaghi et al., 2017). A prolonged PR interval indicates first-degree atrioventricular block, a risk factor for AF (Cheng et al., 2009). P-wave terminal force measures electrical conduction and atrial dysfunction can cause this to elevate (Yaghi et al., 2017). Further, disorders detected using electrocardiogram, such as paroxysmal supraventricular tachycardia and Bayes syndrome, may also indicate AC (Yaghi et al., 2017).

LA fibrosis, which can be detected through cardiac MRI as delayed uptake of gadolinium, is also used as a marker of AC (Siebermair et al., 2017). LA fibrosis is characterized by collagen deposits and disorganized myocytes, resulting in LA dysfunction and arrhythmias such as AF (Nattel, 2017).

Lastly, brain natriuretic peptide (BNP) and cardiac troponin are blood serum biomarkers of cardiac disease that can be used to detect AC (Yaghi et al., 2017). Chronic elevation of troponin and BNP have been associated with worse outcomes such as stroke or death (Hijazi et al., 2014).

## Evidence linking atrial cardiopathy with dementia

Several markers of AC have been associated with dementia risk, including LA enlargement, increased BNP, and electrocardiographic markers, but the relationship is still unclear. AC and cognitive impairment share important risk factors such as age, hypertension, heart failure, diabetes, and obesity. Therefore, confounding variables may account for the increased risk of dementia in patients with AC, and further studies are required to investigate this association. The current evidence linking markers of AC and dementia is presented below.

### Left atrial enlargement

LA size is a known risk factor for cardiovascular events, including AF, stroke, and HF, and may be present in both systolic and diastolic heart dysfunction (Khoo et al., 2011; Menichelli et al., 2020). It can be easily measured through non-invasive tests such as transthoracic echocardiography and is considered an important cardiovascular risk factor. Evidence supporting the direct role for LA enlargement as a cause of cognitive impairment is scarce. A prospective study involving older adults from outpatient cardiology clinics found that LA diameter was independently associated with decreased cognitive function, especially regarding language and memory domains, but no changes in whole brain volume (Alosco et al., 2013). A previous study reported a positive correlation between LA size and white matter hyperintensities, a surrogate marker of cognitive dysfunction in the elderly (Oh et al., 2012).

LA size and function have also been linked to cognitive impairment through diverse mechanisms in patients with HF and AF. LA enlargement directly correlates with left ventricular diastolic dysfunction and chronic LA pressure to volume overload, common findings in HF (Zile et al., 2011). In a study including community-based elderly individuals, LA enlargement and the presence of AF were significantly associated with decreased cognitive function in a cross-sectional analysis; however, with a longitudinal follow-up time of 5 years, only AF was associated with increased cognitive impairment (Zhang et al., 2019).

### Increased brain natriuretic peptide

The relationship between BNP levels (a known marker of AC) and cognitive impairment/dementia has been well evaluated. Broadly, BNP levels have been associated with both cognitive function as well as future risk of dementia. It remains unknown if increased BNP is a marker of dementia risk or has a causative role. BNP may serve as an indicator of cognitive function in individuals with pre-existing cardiovascular disease. In a study including adults greater than 55 years of age with known heart disease, it was found that higher BNP levels are associated with worse cognitive function (Gunstad et al., 2006).

Apolipoprotein-E (APOE) polymorphisms, particularly APOE e4, are significant risk factors for neurological disorders, such as Alzheimer’s disease. There is evidence that APOE e4 may also be a risk factor for cardiovascular disease. Further, APOE e4 carriers have significantly higher BNP levels than non-carriers in individuals with HF (Pasqualetti et al., 2017). In Alzheimer’s disease patients, BNP levels are also higher in those that carry an APOE e4 allele (Begic et al., 2019). Contrarily, healthy individuals that carry at least one APOE e4 allele have significantly lower BNP levels than non-carriers (Begic et al., 2019). This suggests that comorbidities may alter the interplay between APOE e4 status and BNP levels.

The implications for the relationship between BNP levels and cognitive status are not limited to individuals with pre-existing cardiovascular comorbidities. In a population-based study of participants without dementia or cardiovascular disease, higher BNP levels were associated with subclinical brain damage, such as smaller total brain volume (Zonneveld et al., 2017). Also in the general population, within a normal BNP range, higher levels of BNP have been associated with mild cognitive impairment (Kara et al., 2017).

Age is also an important factor when considering the relationship between BNP and cognitive function. In general, BNP levels increase with age, even in individuals without any cardiovascular disease (Redfield et al., 2002). BNP levels have been associated with structural brain changes in both younger and older (>60 years of age) individuals, but the structural changes are accompanied by cognitive deficits only in the older population (Veugen et al., 2018).

Beyond the association between BNP and current cognitive status, it is also a risk factor for future cognitive decline. Multiple longitudinal studies, with follow-up times from 10 to 14 years, found that BNP is an independent risk factor for dementia/cognitive decline (Tynkkynen et al., 2015; Mirza et al., 2016; Nagata et al., 2019; McGrath et al., 2020). Further, when looking specifically at an elderly population greater than 75 years of age, with a 5-year follow-up, BNP was again a predictor of worsening cognitive function (Kerola et al., 2010).

BNP is an established marker of AC; however, it is also a marker of HF and AF, both of which are known risk factors for dementia (Santangeli et al., 2012; Chen et al., 2018; Bailey et al., 2021). Importantly, cognitive function may be further impaired in individuals diagnosed with both HF and AF (Myserlis et al., 2017). The commonality of BNP as a marker of AC, HF and AF, raises the question of whether AC is just a bystander, or is it the shared pathophysiological link between AF, HF, and dementia. There is strong evidence for the association between BNP and cognitive impairment, but further studies are required to tease apart the role of AC in this relationship and to better understand the pathophysiologic mechanisms.

### Electrocardiographic abnormalities

In the last decade, several studies have found abnormal P-wave indices (PWIs) to be independent risk factors for cardioembolic ischemic stroke (Kamel et al., 2015; Maheshwari et al., 2017; Chen and Soliman, 2019). PWIs include several electrocardiographic measures such as P-wave axis, P-wave duration, advanced interatrial block, P-wave area, P-wave dispersion, and P-wave terminal force in V1. Most PWIs require automated software, except for the P-wave axis which is the most reported PWI. Electrocardiographic changes are common in the elderly population, probably reflecting functional and structural changes of the aging heart (Chiao and Rabinovitch, 2015). In a study of 80 centenarians (mean age 101.4 ± 1.5 years), less than 30% had a normal P wave, and almost half of them had an interatrial block (Martinez-Selles et al., 2016).

Although less studied, there may be a relationship between PWIs and cognitive impairment. Data from the community-based cohort Atherosclerosis Risk in Communities (ARIC) Neurocognitive Study with 25-year follow-up and 13,714 participants showed that abnormal PWIs are associated with increased risk of cognitive impairment, independently of the presence of AF and ischemic stroke (Gutierrez et al., 2019). The underlying mechanisms between PWIs and cognitive dysfunction are unknown but may involve subclinical cerebral infarcts and decreased brain perfusion resulting in chronic subcortical ischemia.

Interatrial block on electrocardiogram may also be a valuable marker for different clinical outcomes, including stroke and dementia. Its presence may predict AF, stroke, cognitive impairment, and underlying AC based on atrial remodeling (Bayes de Luna et al., 2017).

The prospective BAYES registry included elderly patients with structural heart disease and absence of AF who were followed for a median of 22 months. Results suggest interatrial block (including both partial and advanced interatrial block) may be a risk factor for cognitive impairment (Martinez-Selles et al., 2020). This finding was also true for patients with mild cognitive impairment (Herrera et al., 2020).

## Pathophysiology of atrial cardiopathy-related cognitive impairment

Observational studies suggest that AC itself (in the absence of HF or AF) is a risk factor for stroke and cognitive impairment (Kamel et al., 2016; Freedman et al., 2020). However, as mentioned in previous sections, the pathophysiology of cognitive dysfunction in patients with AC is unknown and research in this field is highly needed. The most likely mechanism is increased LA thrombogenicity leading to cerebral micro and macroembolism, similar to what has been described in AF (Moretti and Caruso, 2020; Moroni et al., 2020). Additionally, the pathophysiological association between AC and cognitive impairment can be explained by the coexistence of AF and HF. The role of these comorbidities as causes of dementia and cognitive impairment in patients with AC are discussed below.

### Atrial fibrillation

Aging leads to changes in cardiac tissue structure and function (including the LA and LA appendage) and is a leading risk factor for cardiovascular disease (Chiao and Rabinovitch, 2015). Although the exact molecular mechanisms and the clinical consequences of atrial aging are unknown, epicardial fat seems to be an essential source of cytokines stimulating myocardial remodeling through connective tissue proliferation (Pandit et al., 2016). Atrial fibrotic remodeling is a distinctive feature of AC, promoting electrical and autonomic remodeling, thus facilitating the development of both AF and cardioembolism (Jalini et al., 2019; Shen et al., 2019). Chronic inflammatory markers, including CRP, TNF-a, IL-2, IL-6, and IL-8, are increased in patients with AF and may provide an inflammatory background similar to what is seen in other age-related conditions, providing a common mechanistic pathway between AC, stroke, and cognitive impairment (Aviles et al., 2003; Guo et al., 2012; Diener et al., 2019; Forloni, 2020).

AF is a known risk factor for stroke, increasing the risk by a factor of 4–5 (Wolf et al., 1991). While there is a clear relationship between AF and cognitive decline, the literature is inconclusive on whether risk of cognitive impairment is independent of stroke risk in individuals with AF. A meta-analysis reported that AF is associated with cognitive decline in patients with and without stroke (Kalantarian et al., 2013). However, other studies have reported that the AF-related risk of dementia can be explained by brain infarcts, with the majority of infarcts being silent (Sposato et al., 2017; Kühne et al., 2022). AF and dementia share multiple risk factors, and while many studies adjust for these risk factors at baseline, they have short follow-up times and are not always reassessed (Rivard et al., 2022). Further, subclinical AF could be a confounding cause of dementia in patients with AC. Additional investigations, with longer follow-up times and larger cohorts, are still required to determine if there is a causal link between AF and dementia.

Our understanding of the shared patho-mechanisms between AC and cognitive impairment is still developing, but they are likely similar to those that have been implicated in AF. Mechanisms involved in AF, such as inflammation, oxidative stress, and senescence have been extensively reviewed elsewhere (Guo et al., 2021; Sagris et al., 2021; Ihara and Sasano, 2022).

### Heart failure

HF is associated with a 27% increased risk of dementia (Wolters et al., 2018). Low cardiac output in patients with HF can result in reduced cerebral blood flow leading to hypoperfusion of brain structures critical for cognitive function (Adelborg et al., 2017). Impaired vascular autoregulation resulting in white matter injury, neurohormonal dysregulation, systemic inflammation, and cerebral microvascular dysfunction may also contribute to generating a state of chronic cerebral hypoxia leading to brain microinfarcts and neurodegeneration (Adelborg et al., 2017). The latter may be enhanced by oxidative stress, dendritic spine loss, glial activation, and programmed cell death (Jinawong et al., 2021).

Local atrophy of structures involved in memory and other cognitive domains (such as the parahippocampal gyrus) are prominent in patients with HF without clinical dementia (Meguro et al., 2017). This may constitute a treatable and potentially preventable risk factor for cognitive impairment. In a community-based sample of older adults, different left venticle function markers (including echocardiographic parameters and BNP levels) were also associated with structural and functional changes in the brain and not entirely explained by associated risk factors.

Evidence from the ARIC-PET (Atherosclerosis Risk in Communities-positron emission tomography) Study revealed a significant association between florbetapir (a high-affinity beta-amyloid radiotracer) uptake and left ventricular structure and using positron emission tomography (Johansen et al., 2019). Whether these structural and functional changes in heart tissue precede or occur concurrently with amyloid deposition remains unknown.

Atrial dysfunction manifested as HF with preserved ejection fraction may also be linked to cognitive dysfunction. Subclinical ischemic stroke was common in patients with HF with preserved ejection fraction and no prior AF diagnosis and is associated with measurable cognitive deficits in the ARIC cohort (Cogswell et al., 2017). One possible explanation may be undiagnosed paroxysmal AF or AC. AC has also been linked to increased brain amyloid deposition using positron emission tomography, without a similar association in individuals with AF (Johansen et al., 2020).

## Conclusion

Cognitive impairment is a multifactorial disease state with complex interactions between various mechanistic pathways, including neurodegenerative and vascular injury. However, clinical and subclinical cardiac disease may be an important piece of this puzzle and a treatable target for therapeutic intervention. AC may be a substrate for multiple cardiac disorders, such as AF and HF. The association between cognition and both AF and HF is well evaluated but the literature examining AC and cognition is scarce. As our knowledge of AC advances, we will develop a better understanding of AC as a predictor or indicator of cognitive decline. The relationship between AC and dementia may help inform future diagnostic and therapeutic advancements and warrants further investigation. Since there are no proven disease-modifying therapies for dementia, large-scale, multicenter collaborative efforts to evaluate preventive strategies including oral anticoagulants, risk factors modifications, and anti-inflammatory agents are needed.

## Author contributions

All authors listed have made a substantial, direct, and intellectual contribution to the work, and approved it for publication.

## Conflict of interest

The authors declare that the research was conducted in the absence of any commercial or financial relationships that could be construed as a potential conflict of interest.

## Publisher’s note

All claims expressed in this article are solely those of the authors and do not necessarily represent those of their affiliated organizations, or those of the publisher, the editors and the reviewers. Any product that may be evaluated in this article, or claim that may be made by its manufacturer, is not guaranteed or endorsed by the publisher.
